# In the era of monoclonal antibodies targeting the calcitonin gene-related peptide pathway, is it still necessary to stop taking excessive pain medication?

**DOI:** 10.1055/s-0045-1809333

**Published:** 2025-07-28

**Authors:** Renata Gomes Londero

**Affiliations:** 1 Hospital de Clínicas de Porto Alegre, Serviço de Neurologia, Clínica de Cefaleia, Porto Alegre RS, Brazil.

**Keywords:** Headache Disorders, Secondary, Antibodies, Monoclonal, Eptinezumab, Galcanezumab, Fremanezumab

## Abstract

Medication-overuse headache (MOH) was first described in 1951 with ergotamine overuse. Since then, much has been studied about its risk factors, pathophysiology, prevention, and treatment. Despite this, many people still suffer from this condition. Even for those who reach medical care, the path to maintaining significant improvement is neither short nor easy. Here, we propose the ubiquitous individualization of headache treatment. The more we study the condition, the more it becomes evident that different groups of patients benefit from different approaches: starting prophylactic medication immediately or postponing it, providing a bridge treatment or not, and advising patients to either stop medication overuse immediately or reduce it gradually.

Medication overuse headache (MOH) was first described by Peters and Horton in 1951, with ergotamine-overuse. 1 Since then, much has been described about risk factors, pathophysiology, prevention, and treatment. Described in the International Classification of Headache Disorders - 3 (ICHD-3), MOH continues to be a challenge in terms of management, even in the era of monoclonal antibodies targeting the calcitonin gene-related peptide (anti-CGRP mAbs).

In the present study, we propose the ubiquitous individualization of headache treatment. Why is it that the more we study the condition, the more we observe that different groups of patients benefit from different approaches? As in many areas, it is possible to individualize procedures and offer each patient a greater chance of success.

Recent studies show us immediate success rates of 15 to 40% 2 3 in reducing days with headache and use of rescue medication in patients with chronic migraine and MOH between the different forms of management proposed. For many of those patients (20–40%) 4 5 MOH recurs after12 months, in studies that look for those data. Studies of anti-CGRP MAbs raised the possibility of improving patients who were unable to stop MOH on their own. A real-world analysis showed that 60.6% of patients with chronic migraine and MOH had their symptoms resolved after treatment with CGRP antibodies. However, 40% of them did not improve, which can have many explanations.

It has already been observed that the motivation to take pills to treat headache vary in different patients, including relief for pain, fear of having a flare-up after being exposed to conditions known to trigger pain, fear of having a crisis at an important moment in life, and even outright addictive behaviors (some with a history of other substance abuse behaviors).

Besides, early use of migraine-specific preventive treatments (monoclonal antibodies), that could prevent chronification, is limited due to reimbursement and accessibility issues. 6

Understanding the risk factors for MOH can assist in patient management, both seeking to prevent and reverse the situation. There are well-recognized risk factors, broadly studied, including their relative risk (RR): metabolic syndrome (RR = 5.3), use of tranquilizers (5.2), anxiety or depression (4.7), physical inactivity (2.7), use of opioids (2.3), female sex (1.9), low educational level (1.9), chronic musculoskeletal complaints (1.9), age less than 50 years (1.8), and high daily caffeine intake (1.4). 7

There are patients who overuse medications because of other issues, such as low back pain, fibromyalgia, osteoarthritis, orofacial pain etc. It is already known that, in these patients, headaches can persist due to excessive use. Therefore, the management of MOH includes care for patients' other conditions, if any. These patients are not studied, as we know. They are generally excluded from most randomized clinical trials (RCTs), distancing real patients from the results we have.

This data highlights the importance of addressing both the type of primary headache and the specific medications overused, as well as considering individual patient factors such as other pain disorders, psychiatric comorbidities and lifestyle habits, to improve long-term outcomes after detoxification. Keeping these questions in mind can help us treat our patients better.

Scher et al. observed that patients with higher baseline frequency of days using symptomatic medications (> 23 days/month) might have had better outcomes switching them, while the subgroup with lower baseline frequency of days might have had a better outcome without switching. 2

The meta-analysis by Sirilertmekasakul et al. noted that it is possible that overuse of triptans is easier to stop with MABs than overuse of common analgesics or multi-drug (RRs were 1.71, 1.10, and 1.29, respectively). 8

The newest tool in medicine—artificial intelligence—promises to combine the best of evidence-based medicine (facilitating access to good quality information) with individualized care (as each patient will have most of their history registered and available) might allow to offer the best treatment to each patient. 9 Until then, the challenge remains to do so with our oldest tools: listening carefully to the patient, advising them, using what we are capable of learning to individualize care. This will likely result in removing the need for detoxification in some patients, while maintaining it for others.
